# Supplementary material for: Methylglyoxal and D-lactate in cisplatin-induced acute kidney injury: Investigation of the potential mechanism via fluorogenic derivatization liquid chromatography-tandem mass spectrometry (FD-LC-MS/MS) proteomic analysis
Source: PLoS One. 2020 Jul 10;15(7):e0235849. doi: 10.1371/journal.pone.0235849 (PMC7351171; doi:10.1371/journal.pone.0235849)
Supplement: S1 Fig — (PDF) [file pone.0235849.s001.pdf]

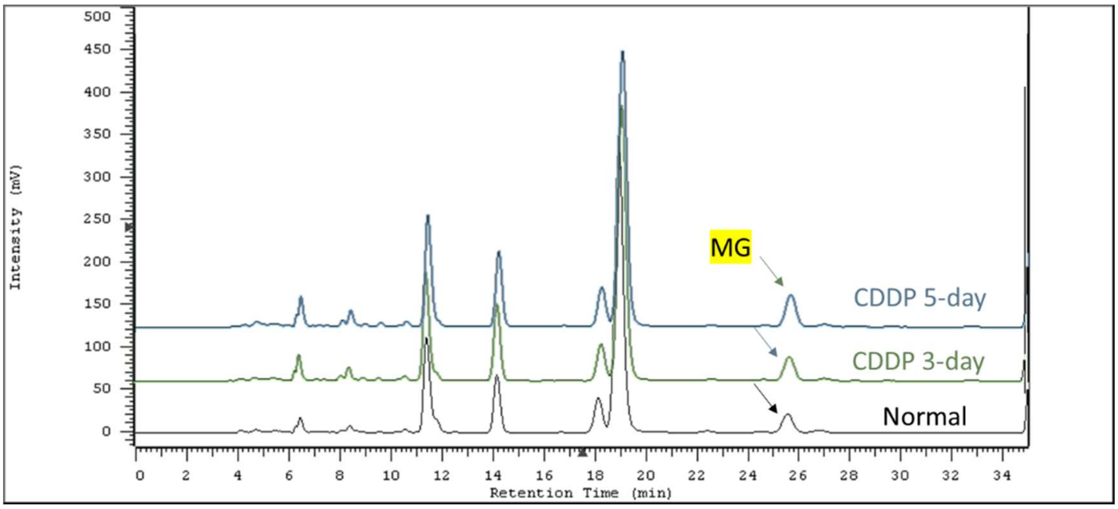

S1 Fig. FD-HPLC chromatograms for the derivatives of methylglyoxal (MG) in the kidney tissues of mice in the control group, CDDP 3-day group and CDDP 5-day group.
