# Supplementary material for: Methylglyoxal and D-lactate in cisplatin-induced acute kidney injury: Investigation of the potential mechanism via fluorogenic derivatization liquid chromatography-tandem mass spectrometry (FD-LC-MS/MS) proteomic analysis
Source: PLoS One. 2020 Jul 10;15(7):e0235849. doi: 10.1371/journal.pone.0235849 (PMC7351171; doi:10.1371/journal.pone.0235849)
Supplement: S1 Table — (PDF) [file pone.0235849.s003.pdf]

S1 Table. Differential proteins identified in the kidney tissues of the CDDP 3-day group vs. control mice.

Peak (Retention time)	Protein Name	MW(Da)	Score	GI NO.
	**Marker**			
26.2	Glial fibrillary acidic protein	48437	45	gi|51066
29.9	Alpha-fetoprotein	47195	48	gi|191765
225	Glial fibrillary acidic protein	48437	56	gi|51066
220	Alpha-fetoprotein	47195	45	gi|191765
	**Respiration**			
155.1	Cytochrome c oxidase	9285	65	gi|31981830
155.1	Hypothetical protein 4732456N10	58230	79	gi|29244176
155.1	Hemoglobin subunit alpha	15076	44	gi|122441
157.9	Alpha-globin	6212	81	gi|193761
157.9	Hemoglobin subunit alpha	15076	80	gi|122441
200.8	Haemoglobin beta-2 chain	16324	182	gi|1183933
200.8	Hemoglobin subunit beta-1	15830	133	gi|122513

200.8	Hemoglobin beta 2	15886	120	gi|187369324
204	Hemoglobin subunit beta-1	15830	101	gi|122513
204	Haemoglobin beta-2 chain	16324	79	gi|1183933
204	Hemoglobin beta	15653	67	gi|229301
212.5	Hemoglobin subunit beta	16046	83	gi|62901559
212.5	Haemoglobin beta-2 chain	16324	61	gi|1183933
215.6	Hemoglobin subunit beta-1	15830	76	gi|122513
218.5	Hemoglobin subunit beta-1	15830	74	gi|122513
231.2	Hemoglobin subunit beta-1	34455	48	gi|1698718
231.2	Glutathione S-transferase mu 1	3242	51	gi|50165
	**Electron-transfer system**			
218.5	Electron transferring flavoprotein β polypeptide	24606	139	gi|38142460
	**Defense**			
44.4	Cu/Zn superoxide dismutase	15752	91	gi|226471
178.8	Cu/Zn superoxide dismutase	15752	78	gi|226471
200.8	Glutathione peroxidase	22276	55	gi|2673845

200.8	Peroxisomal membrane protein 20	17004	76	gi|6746357
204	Peroxisomal membrane protein 20	17004	76	gi|6746357
212.5	Peroxisomal membrane protein 20	17004	183	gi|6746357
215.6	Peroxiredoxin V	21975	48	gi|6644338
218.5	Peroxiredoxin-6	24855	64	gi|3219774
231.2	Catalase	36564	90	gi|10946870
231.2	Aldo-keto reductase family 1, member A4	46555	83	gi|6996911
231.2	Aldo-keto reductase		44	gi|6561420
237.6	Peroxiredoxin 1	22162	41	gi|6754976

Glycolytic system

200.8	Fructose-bisphosphate aldolase B	39548	144	gi|15723268
204	Fructose-bisphosphate aldolase B	39548	66	gi|15723268
212.5	Fructose-bisphosphate aldolase B	39548	227	gi|15723268
215.6	Fructose-bisphosphate aldolase B	39548	62	gi|15723268

Metabolism

51.9	TI-225	14167	86	gi|1167510

231.2	Argininosuccinate synthetase	29993	71	gi|12843914
463.6	Cytosolic malate dehydrogenase	36454	117	gi|387129
	**Other**			
27.4	mCG144996	38119	59	gi|148672085
29.9	mCG144996	38119	61	gi|148672085
29.9	Hypothetical protein 4732456N10	58230	60	gi|29244176
49	mCG144996	38119	77	gi|148672085
116.6	Peptidylprolyl isomerase A	17960	68	gi|6679439
157.9	mCG144996	38119	36	gi|148672085
178.8	Hypothetical protein 4732456N10	58230	59	gi|29244176
204	mCG144996	38119	64	gi|148672085
215.6	Parkinson disease protein 7	20008	68	gi|55741460
215.6	mCG144996	38119	38	gi|148672085
220	mCG144996	38119	52	gi|148672085
234.2	mCG144996	38119	66	gi|148672085
234.2	mKIAA0400 protein	107701	44	gi|50510473
237.6	mCG144996	38119	54	gi|148672085
404.1	mCG144996	38119	63	gi|148672085

404.1	Gamma-actin	40992	38	gi|809561
407	mCG144996	38119	105	gi|148672085
407	Gamma-actin	40992	86	gi|809561
407	put. beta-actin (aa 27-375)	39161	68	gi|49868
407	actin, beta-like 2	41977	44	gi|30425250
471.8	mCG144996	38119	78	gi|148672085
