# Supplementary material for: Methylglyoxal and D-lactate in cisplatin-induced acute kidney injury: Investigation of the potential mechanism via fluorogenic derivatization liquid chromatography-tandem mass spectrometry (FD-LC-MS/MS) proteomic analysis
Source: PLoS One. 2020 Jul 10;15(7):e0235849. doi: 10.1371/journal.pone.0235849 (PMC7351171; doi:10.1371/journal.pone.0235849)
Supplement: S2 Table — (PDF) [file pone.0235849.s004.pdf]

S2 Table Differential proteins identified in the kidney tissues of the CDDP 5-day group vs. control mice.

Peak (Retention time)	Protein Name	MW(Da)	Score	GI NO.
	**Marker**			
35.6	Alpha-fetoprotein	47195	48	gi|191765
	**Other**			
49	mCG144996	38119	77	gi|148672085
	**Metabolism**			
51.9	TI-225	14167	86	gi|1167510
178.8	Hypothetical protein 4732456N10	58230	59	gi|29244176
204	mCG144996	38119	64	gi|148672085
234.2	mCG144996	38119	66	gi|148672085
