# Supplementary figures and images for: Methylglyoxal and D-lactate in cisplatin-induced acute kidney injury: Investigation of the potential mechanism via fluorogenic derivatization liquid chromatography-tandem mass spectrometry (FD-LC-MS/MS) proteomic analysis
Source: PLoS One. 2020 Jul 10;15(7):e0235849. doi: 10.1371/journal.pone.0235849 (PMC7351171; doi:10.1371/journal.pone.0235849)

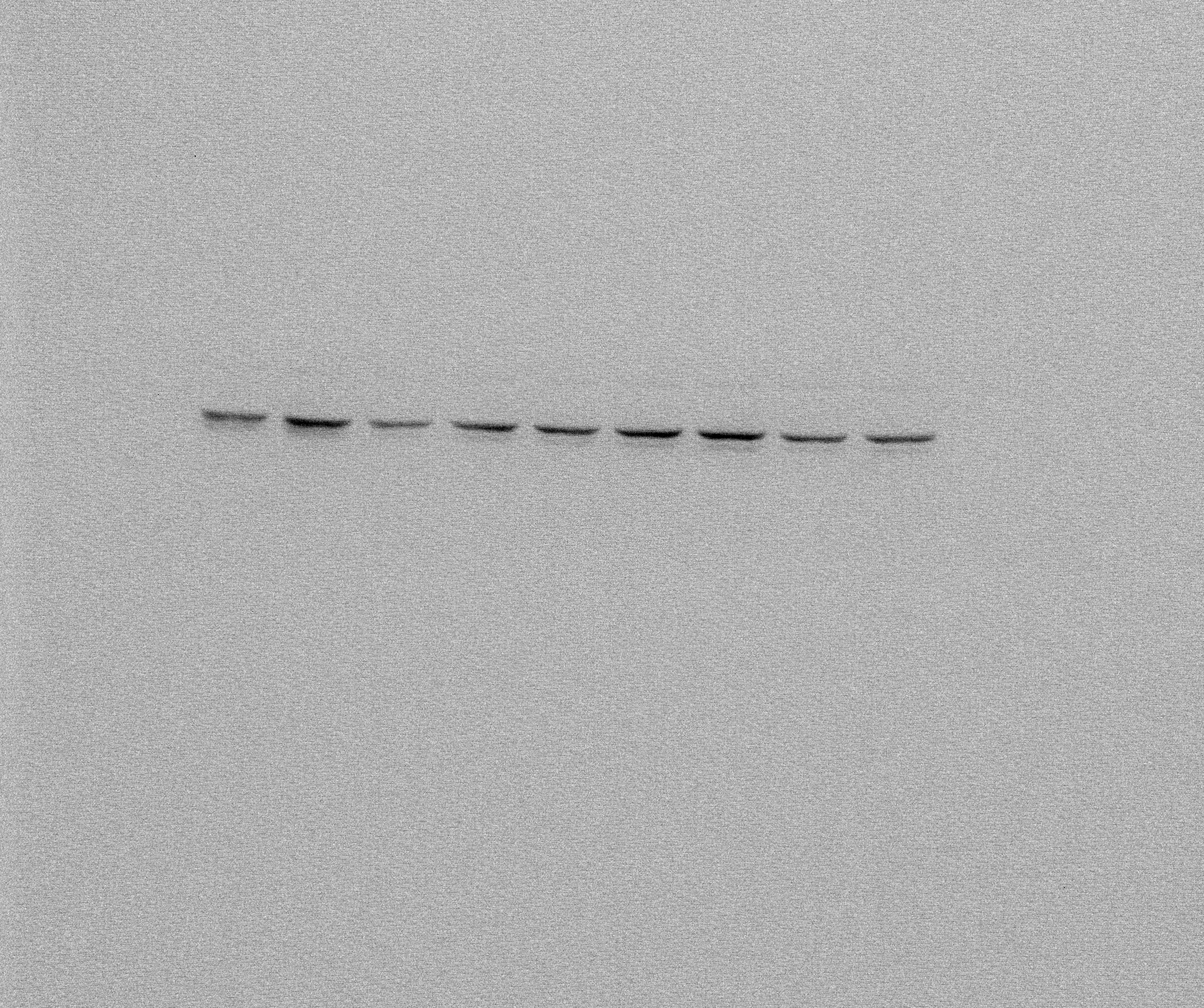

Supplement: S1 Raw Images — (TIF) [file pone.0235849.s005.tif]

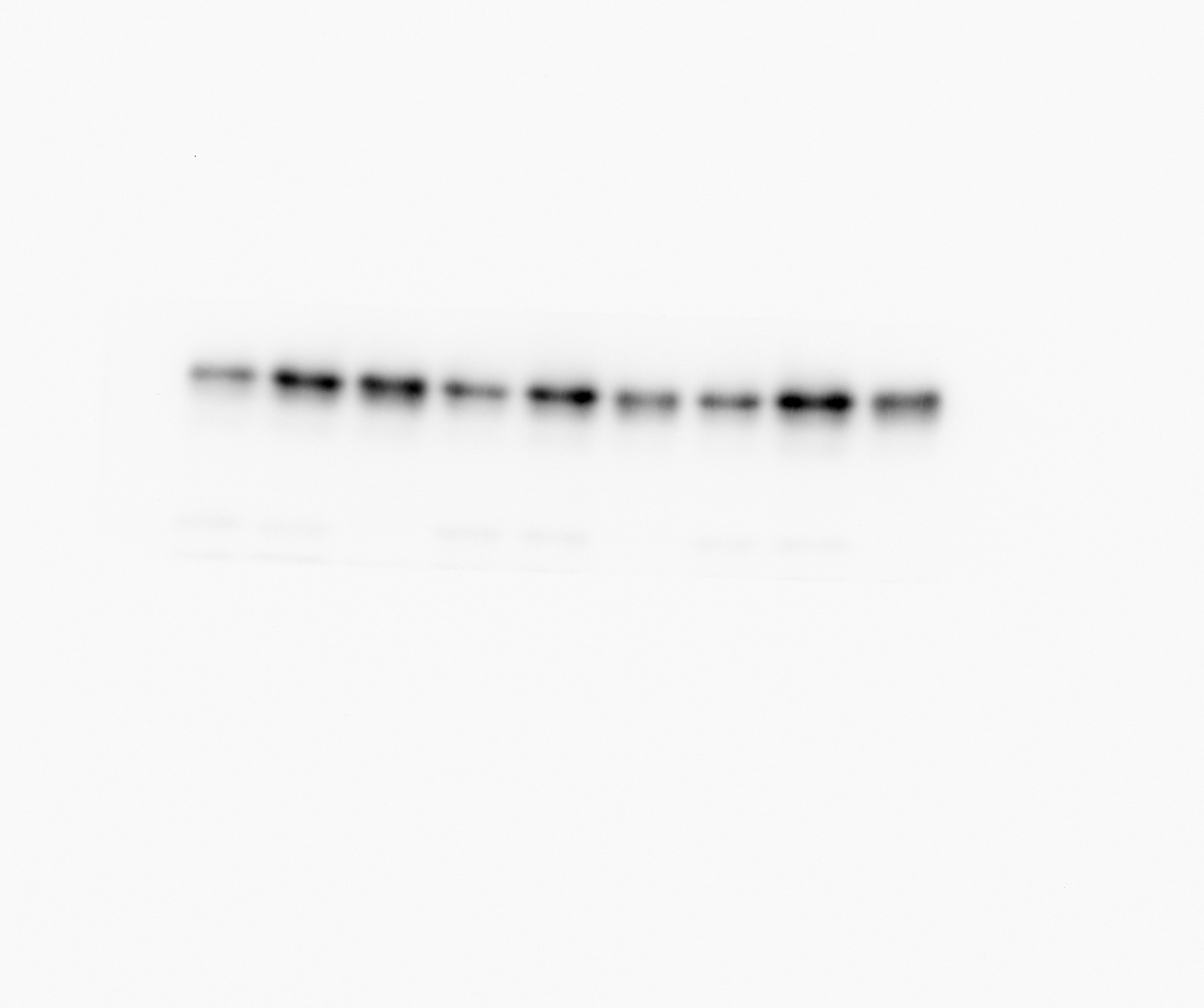

Supplement: S2 Raw Images — (TIF) [file pone.0235849.s006.tif]

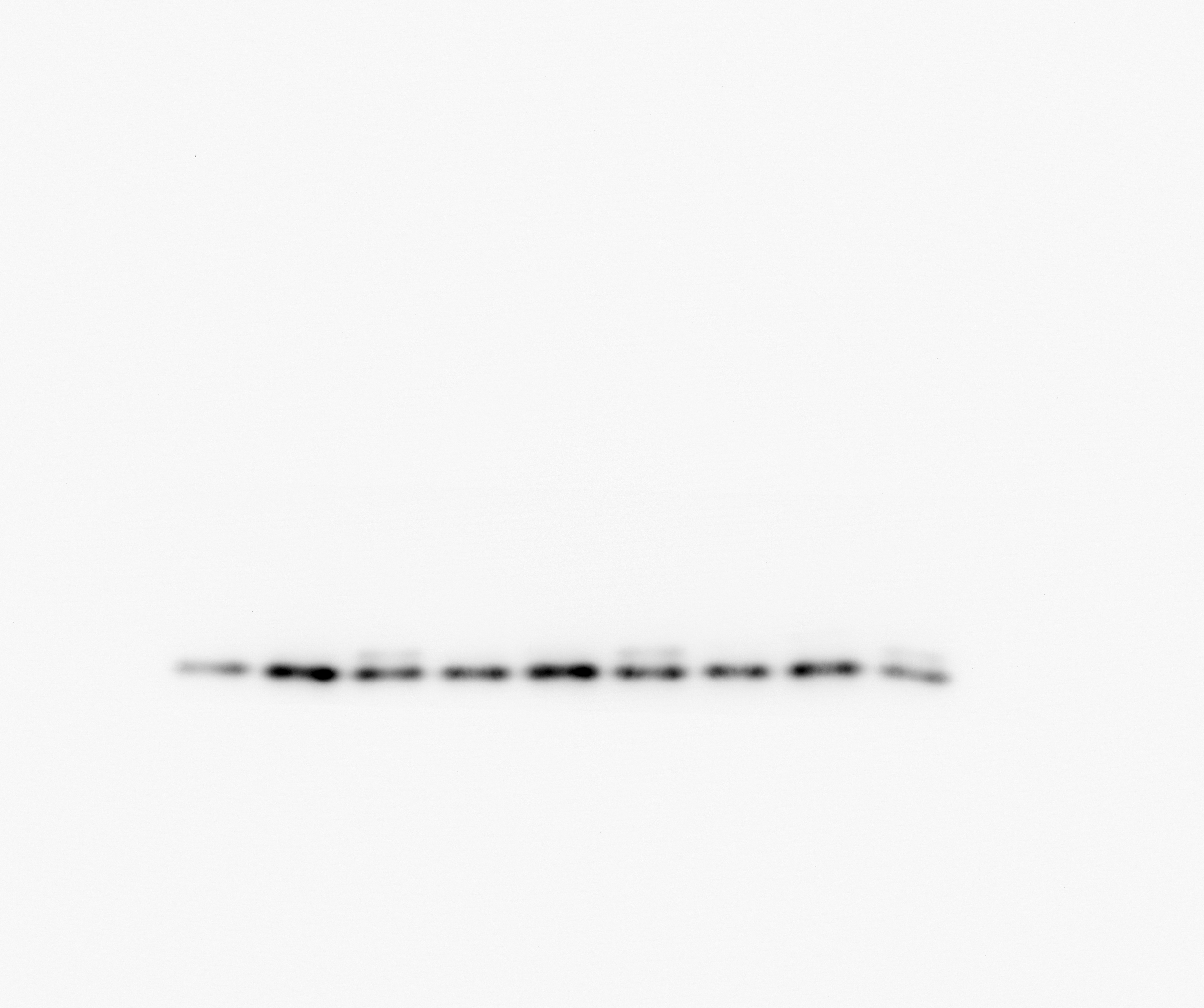

Supplement: S3 Raw Images — (TIF) [file pone.0235849.s007.tif]
