# Supplementary material for: Methylglyoxal and D-lactate in cisplatin-induced acute kidney injury: Investigation of the potential mechanism via fluorogenic derivatization liquid chromatography-tandem mass spectrometry (FD-LC-MS/MS) proteomic analysis
Source: PLoS One. 2020 Jul 10;15(7):e0235849. doi: 10.1371/journal.pone.0235849 (PMC7351171; doi:10.1371/journal.pone.0235849)
Supplement: S1 File — (PDF) [file pone.0235849.s008.pdf]

動物實驗申請表

「本表請留存於貴機構實驗動物照護及使用委員會(或小組)備查，勿須報送本會;惟如使用猿猴、犬、貓進行科學應用時，應提供審核通過之申請表影本列為年度監督報告之附件。」

申請單號: LAC-2019-0167　　　　日期: 2019-04-18

一、　計畫主持人：陳必立 Bi-Li Chen　　職稱：主任　　連絡電話：0970405608

二、　單位：臺北醫學大學附設醫院藥劑部

實驗地點：北醫實驗動物中心　　實驗內容：

三、　計畫/課程/試驗名稱：

(中文) 以尿中甲基乙二醛、D-乳酸評估順氯氨鉑對三種品系小鼠之腎毒性影響

(英文) Evaluation of the Cisplatin Renal Toxicity in Three Strain Mice Using Methylglyoxal and D-lactate in the Urine

計劃類別：藥物及疫苗類

四、　經費來源：臺北醫學大學附設醫院專題研究計畫/自籌

五、　執行期限：2019-05-01 至 2020-05-01

延續型計劃：

六、負責進行動物實驗之相關人員資料:

姓名	職稱	參與實驗期限	具有動物實驗相關技術與經驗年數	佐證資料上傳	附影像光碟
林家宇	組長	2019-05-01 至 2020-05-01	2	P2095_M4091_5cb7dfb5.pdf	

七、實驗所需之動物:

動物別/品系	使用量	動物來源	動物飼養場所	是否需要繁殖
小鼠:小鼠 / Balb/c	10	樂斯科生物科技	北醫實驗動物中心	否
小鼠:小鼠 / C57BL/6	10	樂斯科生物科技	北醫實驗動物中心	否
小鼠:小鼠 / 其他:DBA/2	10	樂斯科生物科技	北醫實驗動物中心	否

註a： 保育類野生動物請加註，並另依野生動物保育法相關規定辦理。

註b： 1.動物來源可能為國內外合法繁殖場(例如國家實驗動物中心，樂斯科生物科技有限公司，美國JAX實驗室・・・等)、其他國內外研究機構之轉讓予贈與(例如美國或歐洲的大學，EMMA・・・等)、小型私人繁殖場及野外捕捉等，請說明動物來源請說明動物來源，再由照護委員會(小組)評估適當性與合法性。

2.自野外捕捉隻動物請加註，並另加說明來源地區、隔離檢疫方式及隔離期間；取自民間市場者，必要時需比照辦理。

註c： 如動物飼養於非本機構之其他場所，需提供該場所所屬機構名稱、地址及該場所核准營運之證明文件(租借場地進行)或審核通過之動物實驗申請表(委託或合作)。

註d： 如需要繁殖「實驗動物(只供應做科學應用目的使用者)」，請填寫附錄一。

八、動物飼養場所類別:

本校動物中心

九、請簡述本研究之目的:

以螢光高效液相層析法，比較三種品系之小鼠對於CDDP誘導腎臟發炎期間尿中甲基乙二醛、D-乳酸的變化，配合臨床檢驗與病理切片。藉此瞭解各品系對CDDP的反應，以及尿液中甲基乙二醛、D-
乳酸在各品系小鼠腎臟發炎所扮演的角色。

十、請以動物實驗應用3Rs之替代及減量原則,說明動物實驗試驗設計、實驗動物需求、動物種別及數量之必要性:

動物實驗申請表

「本表請留存於貴機構實驗動物照護及使用委員會(或小組)備查，勿須報送本會;惟如使用猿猴、犬、貓進行科學應用時，應提供審核通過之申請表影本列為年度監督報告之附件。」

申請單號: LAC-2019-0167　　日期: 2019-04-18

(一)動物科學應用替代方式說明:

1.研究內容:

其他

其他:病理變化及代謝物分析

2.是否進行實驗前替代方式評估?

是 ， 此研究之目的在比較不同種系小鼠對於CDDP引起之腎損傷是否不同，同時分析並比較尿中methylglyoxal及D-lactate之變化。
若以細胞實驗進行，有以下限制
1. CDDP引起之損傷來自DNA損壞、發炎、凋亡等等，且包含不同細胞間的免疫反應，且需以病理切片及臨床數值驗證腎損傷。
2. 評估腎臟損傷程度之尿液分析方法也同時是此研究的重點，因此動物實驗仍有其必要性。
3.使用活體動物之必要性?

已確實了解動物保護法第15條第1項及非活體動物替代方式之相關規範。

已確實遵守3R原則(替代、減量、精緻化)進行實驗設計及實驗前評估。

腎臟整體之發炎、免疫反應，無法以細胞實驗觀察。

(二)法源依據:

(三)參考文獻:"Cisplatin (5 mg/kg body weight) was injected once, 12 h after capsaicin administration for 4 consecutive days. The mice were sacrificed the day after the final cisplatin injection." [Jung SH, Kim HJ, Oh GS, et al., Capsaicin ameliorates cisplatin-induced renal injury through induction of heme oxygenase-1. Molecules and Cells 2014,37(3):234–240.]

"the mice were housed for 12 h (8:00 p.m. to 8:00 a.m.) at room temperature individually in rodent metabolic cages with free access to water for urine collection. Sediment was removed from the urine samples using centrifugation (700 g at 4°C for 5 min). The supernatants were stored at 80°C until urinalysis." [Huang TC, Chen SM, Li YC, Lee JA. Urinary d-lactate levels reflect renal function in aristolochic acid-induced nephropathy in mice. Biomed Chromatogr. 2013 Sep;27(9):1100-6.]
(四)詳細說明動物實驗試驗設計(動物分組方法、每組使用動物數量等):

動物分組：實驗組3組，總共3組
每組使用數量：10隻
總共所需雙數為：3組x10隻/每組=30隻

本研究使用雌性純系小鼠，每5隻關在一個籠子中，動物房為12小時的光照和黑暗交替並維持恆溫。小鼠每日投予CDDP (5 mg/kg/dose/day, i.p.)，連續給與5個劑量。

十一、請以動物實驗應用3Rs之精緻化原則,詳細說明實驗中所進行之動物實驗內容:

(一)實驗物質之投予、採樣方法及頻率:

1. 給予時機：每日一次
2. 劑量：CDDP 5 mg/kg/dose/day, i.p.
3. 期程：連續5天
4. 採樣方法：給藥前day-3、給藥後day3、day5 採集尿液；給藥後day6採腎臟。
實驗中動物之健康變化:

(二)動物之保定、禁食、禁水、限制行動(如代謝籠、跑步機、行為實驗)的方法及時間:

限制行動：代謝籠收集尿液。(給藥前day-3、給藥後day3、day5)共三次，每次12小時。

"the mice were housed for 12 h (8:00 p.m. to 8:00 a.m.) at room temperature individually in rodent metabolic cages with free access to water for urine collection. Sediment was removed from the urine samples using centrifugation (700 g at 4°C for 5 min). The supernatants were stored at 80°C until urinalysis." [Huang TC, Chen SM, Li YC, Lee JA. Urinary d-lactate levels reflect renal function in aristolochic acid-induced nephropathy in mice. Biomed Chromatogr. 2013 Sep;27(9):1100-6.]

(三)麻醉(鎮靜)方法、劑量、投藥、手術方式與麻醉(手術)後的照護:

常用麻醉藥物-1：Isoflurane 劑量：4% 投藥方式：吸入

其他麻醉藥物： 附件 ：

手術方式：無

麻醉（手術）後的照護：

麻醉後放血犧牲。

(四)如何使動物之緊迫或疼痛降至最低(例如使用鎮靜劑或止痛劑、添加環境豐富化物件等，並依疼痛標準級別與實驗目的，描述動物疼痛處理方式):
麻醉後放血犧牲。

疼痛分級：	本實驗所涉及之操作項目
Category C<br><br>1.動物進行不會造成痛苦或緊迫的操作。2.動物進行只造成短暫或輕微痛苦及緊迫的操作。<br>※這些操作不需使用到止痛藥。	• 周邊淺表血管之注射、採血及留置針<br>• 進行AVMA所認可之安樂死操作<br>• 動物安樂死後採取組織<br>• 對動物施用不會顯著增加死亡率的弱毒性物質

(五)實驗預期結束之時機，以及動物出現何種異常與痛苦症狀時提前人道中止實驗:

實驗預期結束之時機（實驗終點）：

給與藥物5個劑量後結束實驗。

動物出現何種異常與痛苦症狀時提前人道中止實驗：

獸醫師意見(人道因素)，體重下降超過15%，無法正常攝食／飲水

十二、請說明實驗結束後動物之處置方式(如復原處置、安樂死、屍體、處理方法、轉讓...等;若為轉讓請提供計劃實驗申請書):
* 安樂死:
* 動物屍體依動物中心規定處理。

動物別/品系	安樂死方式	補充說明
小鼠:小鼠 / Balb/c	物理性安樂死:9.麻醉後放血	
小鼠:小鼠 / C57BL/6	物理性安樂死:9.麻醉後放血	
小鼠:小鼠 / 其他:DBA/2	物理性安樂死:9.麻醉後放血	

十三、有無進行危險性實驗，如生命危險(含感染性物質、致癌藥物)、放射線及化學危險(含有毒物)實驗:

操作場所：

否

(一)實驗之危險性屬於:

1、進行危險物品實驗施用之方法、途徑及場所：

2、針對實驗人員、實驗動物以及飼養環境所採行之保護措施：

3、實驗廢棄物與屍體之處理方式：

(二)如屬生物危險實驗，請陳述：

是否有生命安全委員會之核准資料：無，理由：

(三)如屬放射線或毒性化學危險實驗，請說明本案向主管機關之申請狀況:

無(實驗內容不涉及放射線或毒性化學危險之操作)。

十四、動物實驗人道管理替代、減量及精緻化(3R)說明

(若有申請補助計畫須檢附3R說明時，請填寫說明。)

本研究計畫涉及動物實驗，已考量「替代(Replace)」、「減量(Reduce)」及「精緻化(Refine)」之3R精神，將實驗設計最佳化，並說明如下：

（一）、3R原則

本實驗計畫已經本人及機構內「實驗動物照護及使用委員會(或小組)」詳實審查，無其他替代方案。

本實驗計畫已經本人及機構內「實驗動物照護及使用委員會(或小組)」詳實審查，已使用最少數量動物。

本實驗計畫已經本人及機構內「實驗動物照護及使用委員會(或小組)」詳實審查，已做到精緻化，或動物福利最佳化。包含:

已考慮並要求執行動物疼痛評估

已考慮並要求執行適當減輕動物痛苦的方式(如:設定人道安樂死時機)

（二）、教育訓練

為促進3R精神之落實，本研究實際負責進行動物實驗之相關人員之教育與訓練經歷：

實驗動物人道管理(例如:動物福利、3R原則)。

實驗專業訓練

（三）、使用動物來源：

動物實驗申請表

「本表請留存於貴機構實驗動物照護及使用委員會(或小組)備查，勿須報送本會;惟如使用猿猴、犬、貓進行科學應用時，應提供審核通過之申請表影本列為年度監督報告之附件。」

為確保本研究計劃實驗品質與效益，本實驗之動物來源為：

AAALAC認證繁殖機構

財團法人國家實驗研究院國家實驗動物中心

（四）、監督機制：

為確保實驗品質與效益，本研究激化相關實驗之監督機制為:

「實驗動物照護及使用委員會(或小組)」，隸屬機構層級為校級委員會

召集人職稱教授

已設置專責專職獸醫師，並參與計畫審查及動物照護與管理。

（五）、行政院農業委員會最近一次實地查核本機構「動物科學應用」之評比紀錄：

(請參考附件)

十五　申請人聲明

1.申請人保證以上所填資料完全屬實，因填報不實而生之後果，申請人願負完全之責任。

2.若本委員會或經委員會委託之獸醫師發現本案中實驗動物處於極度不適狀態，基於維護動物福祉之精神，申請人同意立即將實驗動物安樂死。

申請人，是否同意此項聲明? 是，申請人同意此項申明

申請人保證以上所填資料完全屬實，並確認此申請案之執行與運作符合「動物保護法」及相關法規之規定。

動物教育訓練證明

茲助理 **林家宇** 已具備兩年動物實驗經驗，經確認已能操作實驗動物技術，包含：管灌投藥、麻醉、心臟採血、動物犧牲等技術。

計畫主持人：[illegible]

日期：2019. 03. 28

107年度87家受查機構及查核結果一覽表(依機構編號排序)

項次	機構代號	機構名稱	優	良	尚可	較差	動物房舍所屬縣(市)	備註
1	002	國立屏東科技大學			●		屏東縣	
2	006	國立臺灣海洋大學			●		基隆市	
3	007	國立臺灣大學		●			臺北市	
4	014	國立臺北護理健康大學			●		臺北市	
5	016	財團法人國家實驗研究院實驗動物中心		●			臺北市<br>新竹縣	
6	019	高雄醫學大學		●			高雄市	
7	020	衛生福利部國家中醫藥研究所			●		臺北市	
8	022	台灣東洋藥品工業股份有限公司(製劑研發中心)		●			臺北市<br>基隆市	
9	024	佛教慈濟醫療財團法人花蓮慈濟醫院	●				花蓮縣	
10	026	行政院農業委員會畜產試驗所臺東種畜繁殖場	●				臺東縣	
11	029	行政院農業委員會畜產試驗所新竹分所			●		苗栗縣	
12	030	行政院農業委員會畜產試驗所彰化種畜繁殖場		●			彰化縣	
13	032	行政院農業委員會畜產試驗所恆春分所		●			屏東縣	
14	033	行政院農業委員會畜產試驗所花蓮種畜繁殖場			●		花蓮縣	
15	034	國立中正大學			●		嘉義縣	
16	035	嘉藥學校財團法人嘉南藥理大學		●			臺南市	
17	038	慈濟學校財團法人慈濟大學	●				花蓮市	
18	039	行政院環境保護署環境檢驗所	不列入評比				桃園市	
19	042	國立高雄師範大學			●		高雄市	
20	051	臺北醫學大學	●				臺北市	
21	054	長庚醫療財團法人林口長庚紀念醫院	●				桃園市	
22	057	慈濟學校財團法人慈濟科技大學		●			花蓮市	
23	059	東海大學		●			臺中市	
24	062	施懷哲維克生物科技股份有限公司			●		高雄市	
25	066	國立高雄科技大學(楠梓校區)			●		高雄市	

項次	機構代號	機構名稱	優	良	尚可	較差	動物房舍所屬縣(市)	備註
26	067	國立澎湖科技大學			●		澎湖縣	
27	068	財團法人國家衛生研究院	●				苗栗縣<br>臺南市	
28	073	行政院農業委員會農業藥物毒物試驗所	●				臺中市	
29	074	國防醫學院預防醫學研究所		●			新北市	
30	077	國立宜蘭大學			●		宜蘭縣	
31	078	國立清華大學			●		新竹市	
32	080	華達化學製藥廠股份有限公司			●		新北市	
33	087	中國文化大學			●		臺北市	
34	095	永信藥品工業股份有限公司				●	臺中市	
35	104	汎晟藥物研發股份有限公司	●				臺北市	
36	106	國防醫學院		●			臺北市	
37	112	中臺科技大學		●			臺中市	
38	114	靜宜大學			●		臺中市	
39	115	國立中央大學			●		桃園市	
40	120	長庚大學		●			桃園市	
41	121	大仁科技大學			●		屏東縣	
42	123	臺北市立聯合醫院	不列入評比				臺北市	
43	127	國立臺南大學			●		臺南市	
44	133	進階生物科技股份有限公司		●			新北市	
45	138	樂斯科生物科技股份有限公司	●				宜蘭縣<br>臺北市	
46	139	行政院農業委員會水產試驗所	●				基隆市	
47	141	行政院農業委員會水產試驗所東部海洋生物研究中心			●		臺東縣	
48	142	行政院農業委員會水產試驗所海水繁養殖研究中心			●		臺南市	
49	143	行政院農業委員會水產試驗所淡水繁養殖研究中心			●		彰化縣<br>新竹縣	
50	144	行政院農業委員會水產試驗所東港生技研究中心		●			屏東縣	
51	147	國立嘉義大學		●			嘉義市	
52	152	輔仁大學學校財團法人輔仁大學			●		新北市	
53	154	國立高雄第一科技大學	不列入評比				高雄市	

項次	機構代號	機構名稱	優	良	尚可	較差	動物房舍所屬縣(市)	備註
54	159	大豐疫苗科技股份有限公司			●		臺中市	
55	167	長庚醫療財團法人基隆長庚紀念醫院		●			基隆市	
56	171	財團法人工業技術研究院		●			新竹市<br>新竹縣	
57	176	實踐大學			●		臺北市	
58	179	長庚醫療財團法人高雄長庚紀念醫院	●				高雄市	
59	180	長庚學校財團法人長庚科技大學			●		桃園市	
60	181	國立臺灣大學醫學院	●				臺北市	
61	184	輔英科技大學			●		高雄市	
62	188	長庚醫療財團法人嘉義長庚紀念醫院	●				嘉義縣	
63	195	國立暨南國際大學			●		南投縣	
64	205	南臺科技大學		●			臺南市	
65	221	台美檢驗科技有限公司		●			新北市	
66	229	財團法人國家實驗研究院實驗動物中心南部設施		●			臺南市	
67	232	馬偕學校財團法人馬偕醫學院			●		新北市	
68	233	國立虎尾科技大學		●			雲林縣	
69	240	國立體育大學			●		桃園市	
70	241	昌達生化科技股份有限公司		●			新北市<br>臺北市	
71	243	泉盛生物科技股份有限公司	●				臺北市	
72	251	盛德生物科技股份有限公司	不列入評比				新北市<br>臺北市	裁撤<br>107.10.23
73	260	亮宇生物科技有限公司			●		高雄市	
74	265	啟基生技股份有限公司	不列入評比				桃園市	裁撤107.6.6
75	268	豬博士動物科技股份股份公司	●				苗栗縣	
76	269	壽元化學工業股份有限公司				●	嘉義市	
77	271	協宇生物科技股份有限公司			●		新北市	
78	272	康霈生技股份有限公司		●			新北市	
79	273	磁量生技股份有限公司			●		新北市	
80	274	臺灣汎生製藥廠股份有限公司			●		屏東縣<br>高雄市	

項次	機構代號	機構名稱	優	良	尚可	較差	動物房舍所屬縣(市)	備註
81	285	順天醫藥生技股份有限公司		●			臺北市	
82	287	世宬生物科技顧問股份有限公司			●		新北市	
83	288	財團法人國家實驗研究院儀器科技研究中心	不列入評比				新竹縣<br>新竹市	
84	289	啟弘生物科技股份有限公司		●			新北市<br>臺北市	
85	290	心悅生醫股份有限公司			●		新北市	
86	291	聯合生物藥股份有限公司	不列入評比				新竹縣	
87	292	專心動物醫院	不列入評比				臺北市	

107年度87家受查機構及查核結果一覽表(依受查機構所屬縣(市)排序)

動物保護主管機關	機構代號	機構名稱	優	良	尚可	較差	動物房舍所屬縣(市)	備註
臺北市動物保護處	007	國立臺灣大學		●			臺北市	
	014	國立臺北護理健康大學			●		臺北市	
	016	財團法人國家實驗研究院實驗動物中心		●			臺北市<br>新竹縣	
	020	衛生福利部國家中醫藥研究所			●		臺北市	
	022	台灣東洋藥品工業股份有限公司(製劑研發中心)		●			臺北市<br>基隆市	
	051	臺北醫學大學	●				臺北市	
	087	中國文化大學			●		臺北市	
	104	汎晟藥物研發股份有限公司	●				臺北市	
	106	國防醫學院		●			臺北市	
	123	臺北市立聯合醫院	不列入評比				臺北市	
	138	樂斯科生物科技股份有限公司	●				臺北市<br>宜蘭縣	
	176	實踐大學			●		臺北市	
	181	國立臺灣大學醫學院	●				臺北市	
	241	昌達生化科技股份有限公司		●			臺北市<br>新北市	
	243	泉盛生物科技股份有限公司	●				臺北市	
	251	盛德生物科技股份有限公司	不列入評比				臺北市<br>新北市	裁撤<br>107.10.23
	285	順天醫藥生技股份有限公司		●			臺北市	
	289	啟弘生物科技股份有限公司		●			臺北市<br>新北市	
	292	專心動物醫院	不列入評比				臺北市	
	074	國防醫學院預防醫學研究所		●			新北市	
	080	華達化學製藥廠股份有限公司			●		新北市	
	133	進階生物科技股份有限公司		●			新北市	
	152	輔仁大學學校財團法人輔仁大學			●		新北市	
	221	台美檢驗科技有限公司		●			新北市	

動物保護主管機關	機構代號	機構名稱	優	良	尚可	較差	動物房舍所屬縣(市)	備註
新北市政府動物保護防疫處	232	馬偕學校財團法人馬偕醫學院			●		新北市	
	241	昌達生化科技股份有限公司		●			新北市<br>臺北市	
	251	盛德生物科技股份有限公司	不列入評比				新北市<br>臺北市	裁撤<br>107.10.23
	271	協宇生物科技股份有限公司			●		新北市	
	272	康霈生技股份有限公司		●			新北市	
	273	磁量生技股份有限公司			●		新北市	
	287	世宬生物科技顧問股份有限公司			●		新北市	
	289	啟弘生物科技股份有限公司		●			新北市<br>臺北市	
	290	心悅生醫股份有限公司			●		新北市	
基隆市動物保護防疫所	006	國立臺灣海洋大學			●		基隆市	
	022	台灣東洋藥品工業股份有限公司(製劑研發中心)		●			臺北市<br>基隆市	
	139	行政院農業委員會水產試驗所	●				基隆市	
	167	長庚醫療財團法人基隆長庚紀念醫院		●			基隆市	
宜蘭縣動植物防疫所	077	國立宜蘭大學			●		宜蘭縣	
	138	樂斯科生物科技股份有限公司	●				宜蘭縣<br>臺北市	
桃園市政府動物保護處	039	行政院環境保護署環境檢驗所	不列入評比				桃園市	
	054	長庚醫療財團法人林口長庚紀念醫院	●				桃園市	
	115	國立中央大學			●		桃園市	
	120	長庚大學		●			桃園市	
	180	長庚學校財團法人長庚科技大學			●		桃園市	
	240	國立體育大學			●		桃園市	
	265	啟基生技股份有限公司	不列入評比				桃園市	裁撤<br>107.6.6

動物保護主管機關	機構代號	機構名稱	優	良	尚可	較差	動物房舍所屬縣(市)	備註
新竹縣家畜疾病防治所	016	財團法人國家實驗研究院實驗動物中心		●			臺北市<br>新竹縣	
	143	行政院農業委員會水產試驗所淡水繁養殖研究中心			●		彰化縣<br>新竹縣	
	171	財團法人工業技術研究院		●			新竹市<br>新竹縣	
	288	財團法人國家實驗研究院儀器科技研究中心	不列入評比				新竹縣<br>新竹市	
	291	聯合生物藥股份有限公司	不列入評比				新竹縣	
新竹市動物保護及防疫所	078	國立清華大學			●		新竹市	
	171	財團法人工業技術研究院		●			新竹市<br>新竹縣	
	288	財團法人國家實驗研究院儀器科技研究中心	不列入評比				新竹縣<br>新竹市	
苗栗縣動物保護防疫所	029	行政院農業委員會畜產試驗所新竹分所			●		苗栗縣	
	068	財團法人國家衛生研究院	●				苗栗縣<br>臺南市	
	268	豬博士動物科技股份股份公司	●				苗栗縣	
臺中市動物保護防疫處	059	東海大學		●			臺中市	
	073	行政院農業委員會農業藥物毒物試驗所	●				臺中市	
	095	永信藥品工業股份有限公司				●	臺中市	
	112	中臺科技大學		●			臺中市	
	114	靜宜大學			●		臺中市	
	159	大豐疫苗科技股份有限公司			●		臺中市	
彰化縣動物防疫所	030	行政院農業委員會畜產試驗所彰化種畜繁殖場		●			彰化縣	
	143	行政院農業委員會水產試驗所淡水繁養殖研究中心			●		彰化縣<br>新竹縣	
南投縣家畜疾病防治所	195	國立暨南國際大學			●		南投縣	

動物保護主管機關	機構代號	機構名稱	優	良	尚可	較差	動物房舍所屬縣(市)	備註
雲林縣動植物防疫所	233	國立虎尾科技大學		●			雲林縣	
嘉義縣家畜疾病防治所	034	國立中正大學			●		嘉義縣	
	188	長庚醫療財團法人嘉義長庚紀念醫院	●				嘉義縣	
嘉義市政府建設處	147	國立嘉義大學		●			嘉義市	
	269	壽元化學工業股份有限公司				●	嘉義市	
臺南市動物防疫保護處	035	嘉藥學校財團法人嘉南藥理大學		●			臺南市	
	068	財團法人國家衛生研究院	●				苗栗縣<br>臺南市	
	127	國立臺南大學			●		臺南市	
	142	行政院農業委員會水產試驗所海水繁養殖研究中心			●		臺南市	
	205	南臺科技大學		●			臺南市	
	229	財團法人國家實驗研究院實驗動物中心南部設施		●			臺南市	
高雄市動物保護處	019	高雄醫學大學		●			高雄市	
	042	國立高雄師範大學			●		高雄市	
	062	施懷哲維克生物科技股份有限公司			●		高雄市	
	066	國立高雄科技大學（楠梓校區）			●		高雄市	
	154	國立高雄第一科技大學	不列入評比				高雄市	
	179	長庚醫療財團法人高雄長庚紀念醫院	●				高雄市	
	184	輔英科技大學			●		高雄市	
	260	亮宇生物科技有限公司			●		高雄市	
	274	臺灣汎生製藥廠股份有限公司			●		屏東縣<br>高雄市	
屏東縣政	002	國立屏東科技大學			●		屏東縣	
	032	行政院農業委員會畜產試驗所恆春分所		●			屏東縣	
	121	大仁科技大學			●		屏東縣	

動物保護主管機關	機構代號	機構名稱	優	良	尚可	較差	動物房舍所屬縣(市)	備註
府農業處	144	行政院農業委員會水產試驗所東港生技研究中心		●			屏東縣	
	274	臺灣汎生製藥廠股份有限公司			●		屏東縣<br>高雄市	
花蓮縣動植物防疫所	024	佛教慈濟醫療財團法人花蓮慈濟醫院	●				花蓮縣	
	033	行政院農業委員會畜產試驗所花蓮種畜繁殖場			●		花蓮縣	
	038	慈濟學校財團法人慈濟大學	●				花蓮市	
	057	慈濟學校財團法人慈濟科技大學		●			花蓮市	
臺東縣動物防疫所	026	行政院農業委員會畜產試驗所臺東種畜繁殖場	●				臺東縣	
	141	行政院農業委員會水產試驗所東部海洋生物研究中心			●		臺東縣	
澎湖縣家畜疾病防治所	067	國立澎湖科技大學			●		澎湖縣	
