# Supplementary material for: Methylglyoxal and D-lactate in cisplatin-induced acute kidney injury: Investigation of the potential mechanism via fluorogenic derivatization liquid chromatography-tandem mass spectrometry (FD-LC-MS/MS) proteomic analysis
Source: PLoS One. 2020 Jul 10;15(7):e0235849. doi: 10.1371/journal.pone.0235849 (PMC7351171; doi:10.1371/journal.pone.0235849)
Supplement: Attachment — Submitted filename: Response to reviewers.docx [file pone.0235849.s009.docx]

**The rebuttal letter**

First of all, we appreciated both reviewers to point out the blind points that we did not notice or should state more clearly in the beginning. Here are our responses for the specific comments by each reviewer.

**Response comments from Reviewer #1:**

**General comment:**

Overall, this is an interesting manuscript. The rationale and literature review are appropriate. In general, the data support the conclusions reached by the authors. However, there are several points that need to be addressed by the authors. There are a couple of general comments followed by a more specific listing. First, in Figure 1-4 and 5-8, the text and figure caption mention that the comparisons are performed versus a control group. The figures mention a "normal" group. The labeling needs to be consistent with the text of the manuscript. I recommend changing the label on the figures to 'control'. The other general comment has to do with Tables 1 and 2. Table 1 shows a 9.5 hour gradient for FD-HPLC and Table 2 show a 45 min gradient for proteomics analysis. Yet Figure 6, shows chromatograms for the protein separation and the text states "FD-LC-MS/MS chromatograms". Which gradient was used as the time scales are not consistent with the experimental section?

**Response to general comment:**

We appreciate for you kindly remind us the writing mistake. Figure captions had been revised according to your suggestion.

To response the question by regarding to the retention time of protein separation chromatograms (figure 6). Table 1 shows a 570 min gradient for the HPLC protein separation of mice kidney homogenate. Then, the separated sample were analyzed by LC-MS/MS, which using a 45 mins gradient which shows in Table 2. Figure 6. indicate the result of HPLC protein separation, which the scale does correspond with the 570 min gradient program. The retention time in Table 3 is the MSSCOT database comparison results and the peak height in Figure 7 indicate the protein expression in each group. Here we would like to apologize for the low resolution of the picture of chromatogram due to the software issue. We had tried our best to improve the quality of the pictures.

**Specific comments:**

1. **Page 5, line 7: "PAGE" needs to be defined.**

**Response:** The abbreviation had been changed into “Two-dimensional polyacrylamide gel electrophoresis (2D-PAGE)”.

| **Original** | **Revised** |
| --- | --- |
| **Page 5, line 6**  …Two-dimensional PAGE (2D-PAGE) is widely applied in proteomic studies… | **Page 5, line 6**  …Two-dimensional polyacrylamide gel electrophoresis (2D-PAGE) is widely applied in proteomic studies… |

2. **Page 7, lines 2-5: The authors should state that the additional MS and MASCOT details are provided later in the manuscript.**

**Response:** The MASCOT research detail had been added as follow.

| **Original** | **Revised** |
| --- | --- |
| **Page 15, line 18**  …the temperature of the interface heater was 150 °C. The proteins were identified using MASCOT version 2.2, as previously described [24-26]. | **Page 15, line 18**  …the temperature of the interface heater was 150 °C. After detection, peak was led to the MASCOT, which is one of the LC-MS/MS protein identiﬁcation system by Matrix Science Ltd. (www.matrixscience.com/). The MASCOT is one of the most general protein search engine. The proteins were identified using MASCOT version 2.2, NCBInr as the collate database, 0.5 Da as peptide tolerance and MS/MS tolerance, 2+ for the peptide charge. As previous research described [25-27, 30, 31]. |

3. **Page 7, lies 10-12: What are the four animal groups? How many animals per group? need additional detail here.**

**Response:** The sections describe animal grouping had been revised.

| **Original** | **Revised** |
| --- | --- |
| **Page 7, line 10**  …Twenty-four six-week-old female BALB/c mice… | **Page 7, line 10**  **…**Twenty-five six-week-old female BALB/c mice… |
| **Page 7, line 11**  …National Laboratory Animal Center Foundation and randomly allocated to four groups. All animals were allowed to…. | **Page 7, line 11**  **…**National Laboratory Animal Center Foundation and randomly allocated to three groups, control, CDDP administration for 3 days (CDDP 3 days) and CDDP administration for 5 days (CDDP 5 days). Five animals allocated into one group. All animals were allowed to…. |

4. **Page 15, line 19-20: What are the search criteria used?**

**Response:** The MASCOT research detail had been added as follow.

| **Original** | **Revised** |
| --- | --- |
| **Page 15, line 18**  …the temperature of the interface heater was 150 °C. The proteins were identified using MASCOT version 2.2, as previously described [24-26]. | **Page 15, line 18**  …the temperature of the interface heater was 150 °C. After detection, peak was led to the MASCOT, which is one of the LC-MS/MS protein identiﬁcation system by Matrix Science Ltd. (www.matrixscience.com/). The MASCOT is one of the most general protein search engine. The proteins were identified using MASCOT version 2.2, NCBInr as the collate database, 0.5 Da as peptide tolerance and MS/MS tolerance, 2+ for the peptide charge. As previous research described [25-27, 30, 31]. |

5. **Page 19, Figure 1: What is 'n' for each group? This information should be provided all the figures and in the figure captions.**

**Response:** The figure caption had been revised as follow:

| **Original** | **Revised** |
| --- | --- |
| **Page 19, figure1**  Fig 1. Biochemical analysis of CDDP-induced AKI.  (A) Urinary creatinine, (B) NAG activity and (C) BUN in the control group, CDDP 3-day group and CDDP 5-day group. NAG: N-acetyl-acetylglucosaminidase , BUN: blood urea nitrogen, *p < 0.05, Student’s T-test. | **Page 20, figure 1**  Fig 1. Biochemical analysis of CDDP-induced AKI.  (A) Urinary creatinine, (B) NAG activity and (C) BUN in the control group, CDDP 3-day group and CDDP 5-day group. NAG: N-acetyl-acetylglucosaminidase , BUN: blood urea nitrogen, *p < 0.05, Student’s T-test.  N=5 in each group, every experiment was conducted triple repeat. |

Other figures captions had been revised as well.

6. **Page 26, Figure 4 caption: This caption is inadequate. Additional information in needed.**

**Response:** The figure caption had been revised as follow:

| **Original** | **Revised** |
| --- | --- |
| **Page 26, figure4**  Fig 4. Reverse‐phase HPLC quantification of methylglyoxal in the kidney tissues.  *p < 0.05 vs. control group, Student’s T-test . | **Page 26, figure 4**  **Fig 4.** The quantification results of methylglyoxal content in the kidney tissues. *p < 0.05 vs. control group, Student’s T-test . N=5 in each group, every experiment was conducted triple repeat. |

7. **Page 29, Figure 7: Again, the retention times of the identified proteins do not match the gradient given in Table 3.**

**Response:**

Regarding to the retention time of chromatograms. Our answer is in the response to general comments. In brief, Table 1 shows a 570 min gradient program and Figure 6. indicate the result of HPLC protein separation. The retention time in Table 3 is the MSSCOT database comparison results and the peak height in Figure 7 indicate the protein expression in each group.

**Comments from Reviewer #2 and the responses:**

**Comment 1:**

The Figure 2 lacks scale bars and the different groups have different magnification. Not sure if anything can be concluded from them.

**Response to comment 1:**

To add the scale bar to Figure 2, we had obtained a new set of histological sections’ pictures, and rescored the section. The result consists with pervious scoring result. Scale bar had added to the revised figure.

**Comment 2:**

The data in Figure 3 lacks positive and negative control and does not corroborate previous reports on the role of TNFalpha in this model. The according literature is also not mentioned.

**Response to comment 2:**

a) Regarding to the positive or negative control in the immunofluorescence image, we do recognize the importance of the controls, which could provide a clearer and solid evidence to enhance the causality. In this research, immunofluorescence analysis was conducted on the section of mice kidney samples, the negative control could achieve by using TNF-α gene knock-out mice. Considering the weight of benefit using gene mice and to reduce using experimental animals, we did not include negative control into our experiment design. Furthermore, in our previous published article, the immunofluorescence analysis conducted in mice kidney sections did not include negative control, neither (PMID: 31968011). Therefore, we believe without negative control could still represent the result correctly.
b) Our immunofluorescence analysis result of TNF-α revealed the level of TNF-α was correlated with the kidney damage by observing the biomedical analysis and histological images. ROS attributed by methylglyoxal is a key concept in our study (Figure 9). We believed CDDP could increase the level of methylglyoxal and elevate ROS, which trigger the increased production of TNF-α.

**Comment 3:**

The data in Figure 6 is not very clear and needs better representation with clear mass spectra, since derivates are depicted.

**Response to comment 3:**

As the response to Reviewer #1, we apologize for the low resolution of chromatogram due to the software issue. We had tried our best to improve the quality of the pictures and the figure had been revised to improve resolution.

**Comment 4:**

The immunoblot data in Figure 8 does not represent the data shown in the quantification. Also, MW markers should be added.

**Response to comment 4:**

To add the molecule weight marker to the western blot image, we re-conducted gel electrophoresis and semi-quantification. The trend consisted with the previous results. The images had been revised.

**Comment 5:**

Methods: From the methods section regarding the fluorescent derivatization, the experiments cannot be reproduced. It is not clear how proteins were identified since no tryptic digestion step is performed. If this was top down, more information on peptide/protein identification is needed. Like this, bioinformatic analysis is not feasible.

**Response to comment 5:**

Our protein separation method included trypsin digestion step. After the separation by HPLC, 2.5 μL of 10 mM CaCl2, 20 μL of 50 mM NH4CO3 and 2.5 μL trypsin were added to the protein residues and incubated at 37 °C for 2 h for the digestion. And this protein identification method had been published in several articles (PMID: 31968011, PMID: 19714884, PMID: 29088495, PMID: 22972526), we have confidence that this method is dependable.

The MASCOT research detail had been added as follow.

| **Original** | **Revised** |
| --- | --- |
| **Page 15, line 18**  …the temperature of the interface heater was 150 °C. The proteins were identified using MASCOT version 2.2, as previously described [24-26]. | **Page 15, line 18**  …the temperature of the interface heater was 150 °C. After detection, peak was led to the MASCOT, which is one of the LC-MS/MS protein identiﬁcation system by Matrix Science Ltd. (www.matrixscience.com/). The MASCOT is one of the most general protein search engine. The proteins were identified using MASCOT version 2.2, NCBInr as the collate database, 0.5 Da as peptide tolerance and MS/MS tolerance, 2+ for the peptide charge. As previous research described [25-27, 30, 31]. |

**Comment 6:**

Discussion: Very recent cisplatin proteomics studies have been performed. However, these studies are not mentioned and should be used to compare with the authors’ proteomics findings. The following studies were not mentioned: PMID: 25450742, PMID: 30522767, PMID: 31493026, PMID: 31629959, PMID: 17021608PMID: 24265863

**Response to comment 6:**

Here, we are grateful for the reviewer raising the point that we did not attention previously. After we read the references that Reviewer #2 provided, the discussion in the main draft was revised as below, and references provided by the reviewer had been cited.

| **Original** | **Revised** |
| --- | --- |
| **Page 40, line 13**  …Chen et al. (2020) reported inflammatory reaction might enhance glycolysis, leading the content of MG increment, which is one of the by-products from glycolysis [52]. The exact mechanism still need further rigorous investigation. | **Page 40, line 13**  …Chen et al. (2020) reported inflammatory reaction might enhance glycolysis, leading the content of MG increment, which is one of the by-products from glycolysis [52]. Späth et. al. conducted CDDP induced AKI animal model and investigate the altered protein by LC-MS proteomic method and concluded that endogenous fatty acid biosynthesis is a key natural protective mechanism to counteracted CDDP nephrotoxicity [53]. Allaman et. al reported despite glycolysis, lipid metabolism is an endogenous MG source as well [54]. Which could support the MG source in this animal model is mainly endogenous. The exact mechanism still needs further rigorous investigation.  There are several studies focused on CDDP induced kidney damage using proteomic analyzing method, tried to explore the pathological mechanism of CDDP nephrotoxicity and find potential marker. While the model nor the analyte are not consisting [53, 55-59]. This study used mice as experimental animal and liquid chromatography-tandem mass spectrometry (FD-LC-MS/MS) proteomic analysis method and found DJ-1 and SOD-1 were two proteins could support the role of MG in CDDP induced AKI, which may could provide as a foundation for novel treatment or prevention strategy. |
