# Supplementary material for: Methylglyoxal and D-lactate in cisplatin-induced acute kidney injury: Investigation of the potential mechanism via fluorogenic derivatization liquid chromatography-tandem mass spectrometry (FD-LC-MS/MS) proteomic analysis
Source: PLoS One. 2020 Jul 10;15(7):e0235849. doi: 10.1371/journal.pone.0235849 (PMC7351171; doi:10.1371/journal.pone.0235849)
Supplement: Attachment — Submitted filename: Response to reviewers.docx [file pone.0235849.s010.docx]

**The rebuttal letter**

This letter is to response to the issues raised by Reviewer in minor revision.

Again, we are appreciated to Reviewer’s kindly remind us the writing and grammarly mistakes. We had amended the paragraph according to the Reviewer’s suggestion. The following is the response to the comments from Reviewer.

**Comment 1:** Page 7, lines 13-14: The sentence should be written as: "Five animals were allocated into each group."

**Response:** The paragraph had been revised as follow:

| **Previous revision** | **Current revision** |
| --- | --- |
| **Page 7, line 13-14**  … Five animals allocated into one group… | **Page 7, line 10**  … Five animals were allocated into one group… |

**Comment 2:** Page 16, lines 1-5: The paragraph need to be rewritten. Suggestion "After detection, data was submitted to MASCOT, a protein identification program .... MASOCT is widely-used protein identification search engine. Line 5: the word "collate" should be deleted.

**Response:** The paragraph had been revised as follow:

| **Previous revision** | **Current revision** |
| --- | --- |
| **Page 16, line 1-7**  After detection, peak was led to the MASCOT, which is one of the LC-MS/MS protein identiﬁcation system by Matrix Science Ltd. (www.matrixscience.com/). The MASCOT is one of the most general protein search engine. The proteins were identified using MASCOT version 2.2, NCBInr as the collate database,… | **Page 16, line 1-7**  After detection, data was submitted to MASCOT, a protein identification program, which is one of the LC-MS/MS protein identiﬁcation system by Matrix Science Ltd. (www.matrixscience.com/). MASCOT is widely-used protein identification search engine. The proteins were identified using MASCOT version 2.2, NCBInr as the database,… |

**Comment 3:** Figure captions 1, 2, 3, 4, 5 and 8: The words "triple repeat" should be deleted and replaced with 'in triplicate'

**Response:** The figure caption had been revised as follow:

| **Previous revision** | **Current revision** |
| --- | --- |
| **Page 20, line 5 (figure 1.)**  … N=5 in each group, every experiment was conducted triple repeat. | **Page 20, line 5 (figure 1.)**  … N=5 in each group, every experiment was conducted in triplicate. |
| **Page 22, line 6-7 (figure 2.)**  …N=5 in each group, every experiment was conducted triple repeat. | **Page 22, line 6-7 (figure 2.)**  …. N=5 in each group, every experiment was conducted in triplicate. |
| **Page 23, line 4-5 (figure 3.)**  …N=5 in each group, every experiment was conducted triple repeat. | **Page 23, line 4-5 (figure 3.)**  …. N=5 in each group, every experiment was conducted in triplicate. |
| **Page 26, line 3-4 (figure 4.)**  …N=5 in each group, every experiment was conducted triple repeat. | **Page 26, line 3-4 (figure 4.)**  …. N=5 in each group, every experiment was conducted in triplicate. |
| **Page 28, line 4-5 (figure 5.)**  …N=5 in each group, every experiment was conducted triple repeat. | **Page 28, line 4-5 (figure 5.)**  …. N=5 in each group, every experiment was conducted in triplicate. |
| **Page 35, line 5-6 (figure 8.)**  …N=5 in each group, every experiment was conducted triple repeat. | **Page 35, line 5-6 (figure 8.)**  …. N=5 in each group, every experiment was conducted in triplicate. |
